# Pregnancy in Patients With Hereditary Angioedema and Normal C1 Inhibitor

**DOI:** 10.3389/falgy.2022.846968

**Published:** 2022-02-17

**Authors:** Natalia Gabriel, Fernanda Marcelino, Mariana P. L. Ferriani, L. Karla Arruda, Regis A. Campos, Rozana F. Gonçalves, Herberto Chong-Neto, Nelson Rosario Filho, Solange O. R. Valle, Joao B. Pesquero, Anete S. Grumach

**Affiliations:** ^1^Clinical Immunology, Faculdade de Medicina, Centro Universitario FMABC, Santo André, Brazil; ^2^Hospital Regional da Asa Norte (HRAN), Brasília, Brazil; ^3^Ribeirão Preto Medical School, University of São Paulo, Ribeirao Preto, Brazil; ^4^Department of Internal Medicine, Federal University of Bahia, Salvador, Brazil; ^5^Outpatient Clinic, Belo Horizonte, Brazil; ^6^Department of Pediatrics, Federal University of Parana, Paraná, Brazil; ^7^Serviço de Imunologia, Hospital Universitário Clementino Fraga Filho (HUCFF-UFRJ), Rio de Janeiro, Brazil; ^8^Department of Biophysics, Federal University of São Paulo, São Paulo, Brazil

**Keywords:** pregnancy, hereditary angioedema, hereditary angioedema with normal C1 inhibitor, FXII, mutation

## Abstract

**Background:**

HAE with normal C1 inhibitor (HAE-nC1-INH) has been identified as a bradykinin mediated angioedema. Estrogens are one of the main trigger factors. Pregnancy in HAE with C1 inhibitor deficiency showed variable course, however, few reports are available for HAE-nC1-INH. We evaluated the course of pregnancies in women diagnosed with HAE-nC1-INH.

**Methods:**

Women with diagnosis of HAE-nC1-INH according to the following criteria: clinical manifestations similar to HAE-C1-INH, normal biochemical evaluation and family history were included. A questionnaire about pregnancies was applied after consent. Genetic evaluation for known mutations was performed in all patients.

**Results:**

A total of 45 pregnancies occurring in 26 HAE-nC1-INH patients were evaluated (7/26 patients with *F12* variant). Spontaneous abortion was reported in 8/45 (17.8%) pregnancies. Onset of attacks started before the pregnancy in 18/26 patients; during the pregnancy in 2/26; and after the pregnancy in 6/26. HAE attacks occurred in 24/37 pregnancies (64,7%): during the 1st trimester in 41.7%; 2nd trimester in 12.5%; 3rd trimester in 20.8%; 1st and 3rd trimesters in 4.2% and during the whole pregnancy in 20.8%. Among 15/18 patients who had attacks before pregnancy, symptoms persisted with worsening in 9/15; improvement in 4/15; no change in 1/15, and no response in 1/15.

**Conclusions:**

The occurrence of abortion in HAE-nC1-INH was similar to the expected for not affected women. The 1st trimester of the pregnancy was more symptomatic for HAE-nC1-INH women. Considering the strong relevance of estrogens in HAE-nC1-INH, pregnancy could worsen the course of disease.

## Introduction

Hereditary angioedema (HAE) is a rare disease with autosomal dominant inheritance, characterized by recurrent episodes of subcutaneous and sub-mucosal edema attacks. HAE can be classified in HAE with C1 inhibitor deficiency (HAE-C1-INH) or HAE with normal C1 inhibitor (HAE-nC1-INH) (1). In both cases, angioedema occurs due to excessive activation of the plasma contact system, leading to increased levels of bradykinin and, consequently, an increase in vascular permeability and extravasation of fluids to the extravascular environment (2). The clinical manifestations of HAE-nC1-INH include angioedema attacks affecting extremities, face, tongue, genitals, abdomen, and upper airways. Unnecessary abdominal surgeries and asphyxia are severe complications related to inadequate treatment of HAE patients.

HAE-nC1-INH was first described in 2000 after the observation of angioedema without wheals affecting several women in the same family (3). Since then, genetic variants in the genes coding for Coagulation Factor XII (FXII), plasminogen, angiopoetin 1, kininogen 1, myoferlin, and heparan sulfatase have been described in HAE-nC1-INH patients; however, a subset of patients remain who do not have variants identified, a condition designated as HAE-unknown (HAE-U) (4).

In patients with HAE-nC1-INH with *F12* variants, estrogens have an important role. In a series of 57 patients from the French National Center of Reference for Angioedema, estrogen was associated with attacks in 36 of 38 symptomatic patients (5). In 24/36, exacerbation of symptoms occurred during pregnancy or associated with intake of estrogen-containing oral contraceptives (5). Recently, a systematic review identified clinical differentiators for genetic variants of HAE-nC1-INH. The findings reaffirmed the influence of estrogens mainly in FXII-HAE in comparison with other types of HAE-nC1-INH (6).

In women with HAE-C1-INH, pregnancy showed a variable course, worsening in different periods, suggesting that hormonal changes during the gestation were not the only factor influencing its course (7–9). There are only case reports about the pregnancy in HAE-nC1-INH women (10–19). Therefore, considering the close connection with estrogen and limited information on the course of pregnancies in women with HAE-nC1-INH, we aimed to evaluate the gestational period in these patients.

## Methods

We invited women previously enrolled in the Brazilian cohort of patients with diagnosis of HAE-nC1-INH to respond retrospectively about their pregnancies (20). Diagnosis was based on clinical symptoms, normal biochemical tests for HAE, and family history according to criteria established in 2012 (21). Genetic tests looking for variants in *F12, PLG* and *ANGPT1* genes were performed in all HAE-nC1-INH patients. A questionnaire was applied electronically to collect data on clinical characterization (age at onset of symptoms and diagnosis; clinical manifestations 1 year before the pregnancy and during each trimester of pregnancy; triggering factors, frequency, and severity of attacks); prophylactic and on demand treatment before and during the pregnancy and type of delivery. Only patients above 18 years of age were included. Patients presenting comorbidities which could worsen the pregnancy were excluded. Ethical Committee approved the protocol (CAAE: 98089218.4.0000.0082) and patients signed forms authorizing the study before data completion.

## Results

Twenty-six women with 37 pregnancies and 8 spontaneous abortions were enrolled in the study. Mean ages at onset of symptoms, at diagnosis, and at the start of pregnancy were; 34.6 ± 8.78 years; and 27.4 ± 5.28 years of age, respectively. Two out of eight abortions occurred in diabetic patients. *F12* mutation was identified in 7/26 (26.9%) women, and no patients with PLG or ANGPT1 mutation were detected. The development of symptoms occurred before the first pregnancy in 18/26 patients (mean age: 17.5 years old); during the first pregnancy in 2/26 (mean age 23.5 years old), and 6/26 after the pregnancy (mean age 26.2 years old). Among the six patients who developed symptoms after the first pregnancy, the mean time to onset of symptoms was 2.5 years. There was no difference in age at onset of symptoms according to presence of *F12* variant (19.8 ± 6.91 vs. 20.2 ± 5.95 years in the whole group).

From 37 analyzed pregnancies, 24 were characterized by angioedema attacks and 13 remained asymptomatic. During the pregnancy, attacks affected, preferentially, extremities in 16/24 (66.7%), abdomen in 10/24 (41.7%), and upper airways in 9/24 (37.5%). Besides abdominal pain, gastrointestinal symptoms including nausea in 7/10; vomiting in 6/10; abdominal distension in 5/10, diarrhea in 5/10, and cramps in 3/10 were also described. There was also the involvement of arms 7/24 (29.2%), legs 6/24 (25%), face 9/24 (37.5%), 2/24 neck (8.3%), tongue 4/24 (16.7%), lips 7/24 (29.2%), eyelids 7/24 (29.2%), and genitals 5/24 (20.9%). Additional symptoms referred during the attacks were arthralgia (11/24), headache (8/24), and difficulty urinating (1/24) (Table 1).

**Table 1:** Sites affected by HAE attacks before and during the pregnancy.

**Sites**	**HAE-Unknown** ***n*** **=** **20 (%)**		**HAE-FXII** ***n*** **=** **4 (%)**	
	**Before pregnancy**	**During pregnancy**	**Before pregnancy**	**During pregnancy**
Extremities	12 (80%)	8 (53.3%)	4 (100%)	2 (50%)
Arms	4 (26.7%)	3 (20%)	2 (50%)	2 (50%)
Legs	5 (33.3%)	3 (20%)	2 (50%)	2 (50%)
Face	13 (86.7%)	7 (46.7%)	4 (100%)	0
Neck	4 (26.7%)	2 (13.3%)	1 (25%)	0
Tongue	6 (40%)	2 (13.3%)	0	0
Lips	13 (86.7%)	5 (33.3%)	4 (100%)	0
Eyelids	8 (53.3%)	5 (33.3%)	3 (75%)	0
Genitals	6 (40%)	3 (20%)	3 (75%)	2 (50%)
Abdomen	8 (53.3%)	8 (53.3%)	2 (50%)	3 (75%)
Larynx	5 (33.3%)	4 (26.7%)	1 (25%)	0

Triggering and/or worsening factors included emotional distress 20/23 (87%), trauma 17/23 (73.9%), infectious disease 6/23 (26%), cold weather 2/23 (8.7%), drugs (1/23) (4.3%), unknown 4/23 (17.4%), and no triggering factor identified in 1/23 patients (4.3%). One patient did not respond to this question.

**Figure 1:**
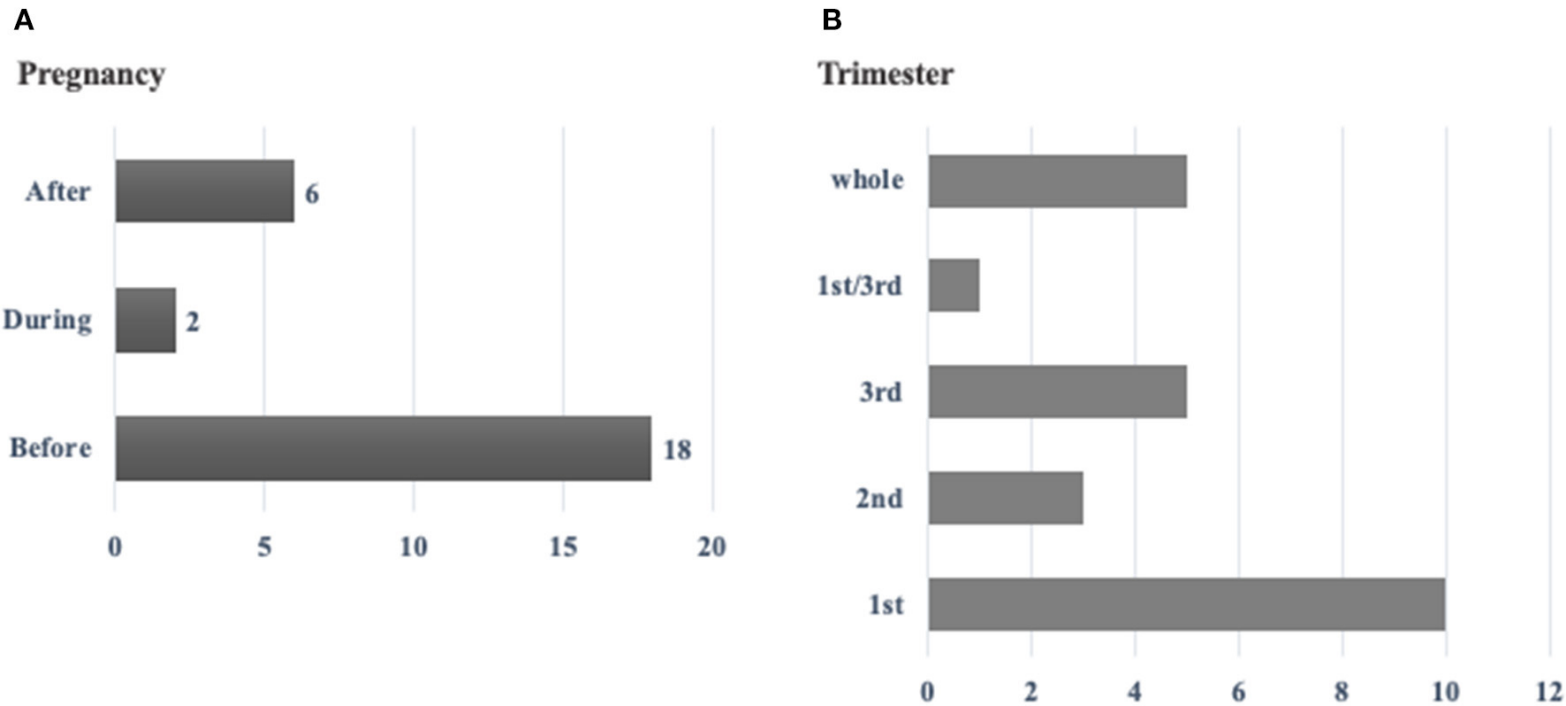
**(A)** Development of HAE symptoms in 26 pregnant women before, during or after the pregnancy*. **(B)** Occurrence of HAE attacks in 24 pregnant women with HAE-nC1-INH according to the period of the pregnancy**. *Before pregnancy corresponds to the year before getting pregnant. During the pregnancy corresponds to the total period of pregnancy. After the pregnancy corresponds to the period of 6 months after delivery. **Whole period means that the women presented attacks during the whole pregnancy.

Regarding timing of attacks during the pregnancy, the occurrence of attacks was in the first trimester in 10/24 (41.7%), second trimester in 3/24 (12.5%), third trimester in 5/24 (20.8%), first and third trimester in 1/24 (4.2%) and during the whole pregnancy in 5/24 (20.8%) (Figure 1). Fifteen patients had attacks both before and during the first pregnancy, which allowed us to make comparisons. Frequency of attacks worsened in 9/15 (60%), improved in 4/15 (26.7%), had no change in 1/15 (6.7%) and 1/15 (6.7%) did not respond. Also, 10/15 (66.7%) reported the same intensity of attacks, 3/15 (20%) reported higher intensity and 2/15 (13.3%) reported lower intensity. Concerning localization of attacks, extremities and abdomen were most affected 8/15 (53.3%) and involvement of upper airways was reported by 4/15 (26.7%).

Among patients with *F12* variant, 10 pregnancies from 7 women were analyzed. Attacks occurred before the pregnancy in 5/7 (71.4%) and after the first pregnancy in 2/ 7 patients (28.6%). Attacks occurred in 4/10 (40%) pregnancies, and 6/10 (60%) remained asymptomatic. Abdomen was affected in 2/4 (50%), extremities, legs, arms and genitals were affected in 2/4 (50%). Two patients had more attacks during the first trimester, one during the first and the third trimesters, and one during the whole pregnancy.

## Discussion

HAE-nC1-INH, first described in 2000, has been associated with variants in six genes, however, a subset of patients remains without a variant identified by genetic analysis, comprising the group designated as HAE-unknown (HAE-U). Only *F12* variants have been identified in Brazilian patients with HAE-nC1-INH so far, predominantly the missense c.983C>A (p.Thr328Lys) variant, found in 132/196 patients and relatives; and the c.971_1018D24del72 deletion which was found in only 2 patients (14, 20). Estrogen exposure related to use of estrogen-containing oral contraceptives [OC], menstruation, pregnancy, and hormone replacement therapy [HRT]) represents an important trigger or aggravating factor in HAE-FXII (22–24). This hormonal influence has not been observed in HAE-PLG patients (6). Our study evaluated pregnancies in women with unknown variants and HAE-FXII, identified in 20% of the patients (25, 26).

The hormonal changes of pregnancy worsened the symptoms in about one third of our patients with HAE-C1-INH previously described (9). Estrogens can interact with most of the steps of the cascade generating bradykinin (2). The hormonal influence could partially explain the symptoms, and some women even report the onset of symptoms during their first pregnancy as it was reported by two patients of our cohort (27). However, the pathophysiology HAE-nC1-INH is not fully understood (24).

Previous reports revealed a variable course of HAE-C1-INH in pregnancy. Intensity and frequency of attacks may worsen, get better or remains the same (7–9, 23). Regarding pregnancies of women with HAE-nC1-INH, only case reports have been published and most of them reported worsening of symptoms during pregnancy (10–13, 15, 28). Although estrogens may increase severity of HAE-nC1-INH, the clinical expression is variable during pregnancy, even for the same patient (5). Our study revealed that the first trimester was the most challenging, with more attacks (41.7%), followed by the third trimester (20.8%). In 5 women, attacks were present during the whole pregnancy. This observation is similar to the published experiences in HAE-C1-INH, however, we had more frequent aggravated symptoms in the second trimester for pregnancies in HAE-C1-INH (7–9). Lower concentrations of C1-INH have been reported in HAE-nC1-INH during the pregnancy and therefore, the occurrence of symptoms could be similar in both situations (5). The difference between HAE-C1-INH and HAE-nC1-INH in our experience could be related to the higher hormonal influence in the second group or the facilitated access to the therapy afterwards. In addition, higher severity of attacks has been described more often in women with HAE-FXII, probably related to higher estrogen sensitivity in comparison with women with HAE-U (10).

Mechanical traumas due to the uterus growth and fetal movements were associated with abdominal attacks in HAE-C1-INH (8, 29), This association was also observed in HAE-nC1-INH (10, 11, 15). According to this theory, a higher frequency of abdominal attacks would be expected in the third trimester; however, symptoms were predominant in the first trimester in pregnancies of our cohort. It is important to emphasize that an HAE attack during the pregnancy may be misdiagnosed, and other obstetrical complications should be excluded (22). Experimental work in rats showed that bradykinin may increase uterus' contractility (30).

Subcutaneous edema affecting extremities and face predominated in the present study, as previously reported for HAE-C1-INH (9). However, upper airway obstruction was also reported by our patients, which could be of risk considering the reduced number drugs approved for gestational period. The emotional distress was reported as trigger factor by most of the women and the possibility of severe attacks had probably led to this consequence.

Although pregnancy was associated with onset and worsening of symptoms of HAE, few obstetric complications were reported by our patients. Severe complications, including fetal and neonatal death, have been previously reported in patients with HAE (5, 11). Recently, recurrent pregnancy loss was associated with MTHFR mutation in a patient with HAE-nC1-INH (18). We reported the follow up of a pregnancy in a patient with HAE-C1-INH and thrombophilia with good outcome (31). In the present study, the occurrence of spontaneous abortion was similar to the expected for non-affected women (25, 26), in contrast with our previous description in HAE-C1-INH pregnancies (9).

Pregnant patients with HAE should be assisted carefully, since the therapeutic options are limited during pregnancy, and experience with HAE-nC1-INH treatment is restricted to case reports or small case series. Two of our patients used pdC1-INH during the attacks and another one treated with FFP. Tranexamic acid was previously prescribed for HAE-C1-INH (9) and no pregnancies received prophylactic therapy in this cohort of HAE-nC1-INH. A management plan should be coordinated by an HAE specialist, with C1-INH concentrate available either in the maternity center or at home, for initiation of therapy as early as possible (22). The use of pdC1-INH during the pregnancy of HAE-nC1-INH women as long-term prophylaxis has been described without further complications (16). In Brazil, the access to pdC1-INH is restricted and it is not included among high-cost drugs which are provided by the government (31).

Clinical course, therapeutic options, outcomes, need for a close follow up with an HAE specialist, should be discussed in detail with women with HAE-nC1-INH who are pregnant or who want to become pregnant. A multidisciplinary approach, involving the obstetrician and other health care professionals when needed, would be beneficial. In addition, genetic counseling should be provided. Pregnancy could not be inputted as more dangerous for women with HAE-nC1-INH than the disease *per se*. In the present study, few women presented HAE-FXII, limiting the conclusion about a different course of the pregnancy in comparison with those with HAE-U, however, a strong relevance of estrogens in HAE-FXII has been observed. Prospective studies to assure appropriate management of HAE-nC1-INH in women during pregnancy are necessary.

## Data Availability Statement

The original contributions presented in the study are included in the article, further inquiries can be directed to the corresponding authors.

## Ethics Statement

The studies involving human participants were reviewed and approved by Ethical Committee of Centro Universitario FMABC. The patients/participants provided their written informed consent to participate in this study.

## Author Contributions

NG and AG contributed to the study conception and design. The first draft of the manuscript was written by NG, LA, and AG. All the authors contributed to data collection and read and approved the final manuscript.

## Funding

LA was awarded productivity grant from National Council for Scientific and Technological Development (CNPq) (306702/2019-3). AG awarded productivity grant from National Council for Scientific and Technological Development (CNPq) (308556/2018-6).

## Conflict of Interest

The authors declare that the research was conducted in the absence of any commercial or financial relationships that could be construed as a potential conflict of interest.

## Publisher's Note

All claims expressed in this article are solely those of the authors and do not necessarily represent those of their affiliated organizations, or those of the publisher, the editors and the reviewers. Any product that may be evaluated in this article, or claim that may be made by its manufacturer, is not guaranteed or endorsed by the publisher.

## Abbreviations

ANGPT1: angiopoetin 1
FXII: Coagulation Factor XII
FXII-HAE: HAE with FXII mutation
PLG: plasminogen
HAE: Hereditary Angioedema
HAE-C1-INH: HAE with C1 inhibitor deficiency
HAE-nC1-INH: HAE with normal C1 inhibitor
HAE-U: HAE-unknown.
